# Glycaemic control for patients with severe acute brain injury: Protocol for a systematic review

**DOI:** 10.1111/aas.14166

**Published:** 2022-11-12

**Authors:** Anne‐Sophie Worm Fenger, Markus Harboe Olsen, Maria Louise Fabritius, Christian Gunge Riberholt, Kirsten Møller

**Affiliations:** ^1^ Department of Neuroanaesthesiology, The Neuroscience Centre Copenhagen University Hospital—Rigshospitalet Copenhagen Denmark; ^2^ Department of Neurorehabilitation, Traumatic Brain Injury, The Neuroscience Centre Copenhagen University Hospital—Rigshospitalet Copenhagen Denmark; ^3^ Department of Clinical Medicine, Faculty of Health and Medical Sciences University of Copenhagen Copenhagen Denmark

**Keywords:** acute brain injury, glycaemic control, intensive care

## Abstract

**Background:**

Hyperglycaemia is common in patients with acute brain injury admitted to an intensive care unit (ICU). Many studies have found associations between development of hyperglycaemia and increased mortality in hospitalised patients. However, the optimal target for blood glucose control is unknown. We want to conduct a systematic review with meta‐analysis and trial sequential analysis to explore the beneficial and harmful effects of restrictive versus liberal glucose control on patient outcomes in adults with severe acute brain injury.

**Methods:**

We will systematically search medical databases including CENTRAL, Embase, MEDLINE and trial registries. We will search the following websites for ongoing or unpublished trials: http://www.controlled-trials.com/, http://www.clinicaltrials.gov/, www.eudraCT.com, http://centerwatch.com/, The Cochrane Library's CENTRAL, PubMed, EMBASE, Science Citation Index Expanded and CINAHL. Two authors will independently review and select trials and extract data. We will include randomised trials comparing levels of glucose control in our analyses and observational studies will be included to address potential harms. The primary outcomes are defined as all‐cause mortality, functional outcome and health‐related quality of life. Secondary outcomes include serious adverse events including hypoglycaemia, length of ICU stay and duration of mechanical ventilation, and explorative outcomes including intracranial pressure and infection. Trial Sequential Analysis will be used to investigate the risk of type I error due to repetitive testing and to further explore imprecision. Quality of trials will be evaluated using the Cochrane Risk of Bias tool, and quality of evidence will be assessed using the Grading of Recommendations, Assessment, Development and Evaluations (GRADE) approach.

**Discussion:**

The results of the systematic review will be disseminated through peer‐reviewed publication. With the review, we hope to inform future randomised clinical trials and improve clinical practice.

## 1 INTRODUCTION

Hyperglycaemia is common in critically ill patients, even in those without diabetes mellitus and is associated with high morbidity and mortality.^1^, ^2^, ^3^ It is thought to be generated by the inflammatory stress response causing release of cortisol, catecholamines and cytokines. In turn, this response triggers both increased gluconeogenesis and peripheral insulin resistance.^4^, ^5^ Fasting or treatment with parenteral nutrition may exacerbate these effects by bypassing the incretin effect.^6^, ^7^ Treatment of hyperglycaemia with insulin administered as bolus injection or infusion may reduce morbidity and mortality in critically ill patients, but the optimal blood glucose target remains unclear.^8^ Several randomised trials have investigated safety and efficacy of tight glycaemic control in intensive care therapy without a precise conclusion.

According to a randomised trial done by Van den Berghe et al.^2^ comparing strict and liberal blood glucose control in surgical patients (primarily cardiothoracic surgical patients) admitted to the intensive care unit (ICU), stricter control (defined as a blood glucose of 4.4–6.1 mmol/L) resulted in a reduction in mortality compared to a liberal regime (defined as a blood glucose of 10–11.1 mmol/L). These results led to a paradigm shift towards strict blood glucose control in patients admitted to ICUs worldwide, and a recommendation of aiming towards a stricter control of blood glucose, although mixed results were found in different populations; Medical ICU patients only showed benefit from strict control with an ICU stay at 3 days or longer, and the length of ICU stay was not predictable,^9^ paediatric patients had a lower mortality in favour of strict control,^10^ and neurological patients admitted to an ICU showed lower morbidity in favour of strict control, but a subgroup of patients with isolated brain injury had no effect on mortality.^11^

The subsequent much larger NICE‐SUGAR trial changed practice with the finding of a strict glucose control (defined as a blood glucose of 4.5–6 mol/L) increased both mortality and the risk of severe hypoglycaemia compared to liberal control (defined as a blood glucose of 7.7–10 mmol/L).^12^ Current general ICU management regimens favour a liberal blood glucose target. Thus, it is common to apply a glucose target between 6 and 10 mmol/L using intravenous insulin infusion or bolus injection and frequent blood glucose measurement to prevent hypoglycaemia.^13^

Patients with severe acute brain injury, in the present context defined as intracerebral haemorrhage (ICH), spontaneous aneurysmal subarachnoid haemorrhage (SAH), traumatic brain injury (TBI), acute ischaemic stroke and anoxic brain injury have a high morbidity and mortality.^14^, ^15^, ^16^, ^17^, ^18^ The aim of intensive care in these patients is to minimise the consequences of the primary brain injury, which is considered to be irreversible, and to avoid secondary brain injury, which is considered to be preventable or reversible and related to stressors such as hypoxia, hypotension, hyperthermia and inflammation.^19^

While hyperglycaemia in patients with acute brain injury patients has been associated with worse outcomes, it is uncertain whether hyperglycaemia is a mediator of secondary brain injury, a marker for severity of critical illness or a compensatory mechanism to allow for a sufficient glucose supply to the injured brain.^13^ The brain uses glucose as its main source of energy, its glucose uptake is not dependent on insulin, and the brain is vulnerable to brief periods with reduced blood delivery of both glucose and oxygen.^20^ In patients with traumatic brain injury, an association between systemic glucose and available brain glucose has been found by both microdialysis studies and PET‐scan studies (positron emission tomography scan).^21^, ^22^ Thus, it is possible that critically ill patients with acute brain injury could benefit from glycaemic targets other than those applied to critically ill non‐brain‐injured patients.

Existing guidelines on glycaemic management are based on outcomes such as mortality and ICU admission time.^12^, ^23^ However, because many patients with acute brain injury survive with an unfavourable outcome, treatment and guidelines concerning these patients may benefit from evidence focusing on both mortality, functional outcome and quality of life.

## 2 WHY IS IT IMPORTANT DO TO THIS REVIEW?

The ideal control of blood glucose levels in critically ill patients is still under debate. It is uncertain whether the guideline used for standard care in intensive care units is transferable to patients suffering from acute brain injury in need of specialised intensive care. A systematic review regarding optimal glycemic control in a broad neurocritical care population was last published in 2012^13^ and since then, additional data from randomised clinical trials comparing glycaemic levels have become available.^24^, ^25^ We intend to include all data possible and to do a Trial Sequential Analyse (TSA) to evaluate if a proper amount of data is available to make a conclusion.

Here, we describe a protocol for a systematic review of randomised clinical trials assessing the beneficial and harmful effects of blood glucose control in patients with severe acute brain injury.

## 3 METHODS AND ANALYSIS

### 3.1 Review questions

- In patients in the intensive care unit with acute brain injury (P), does restrictive glycaemic control (I) compared with liberal glycaemic control (C) change the all‐cause mortality, the functional outcome and health related quality of life (primary outcome)?
- In patients in the intensive care unit with acute brain injury (P), does restrictive glycaemic control (I) compared with liberal glycaemic control (C) change serious adverse events, length of stay in the ICU, proportion of patients with hypoglycaemia, infectious complications or duration of mechanical ventilation (secondary outcomes)?

### 3.2 Search methods

#### 3.2.1 Searches

We will search for published records through Medical Literature Analysis and Retrieval System (MEDLINE), Excerpta Medica database (Embase), Cochrane Central Register of Controlled Trials (CENTRAL), CINAHL, Latin American and Caribbean Health Sciences Literature (LILACS) and Web of Science.

Ongoing or unpublished trials will be searched through the following trial registers: clinicaltrials.org; EU Clinical Trial Register; Chinese Clinical Trial Registry (ChiCTR); International Standard Randomised Controlled Trial Number (ISRCTN) registry; Pan African Clinical Trials Registry (PACTR); Australian New Zealand Clinical Trials Registry (ANZCTR); Clinical Trials Registry—India (CTRI) and the World Health Organization (WHO) International Clinical Trials Registry Platform (ICTRP).

Furthermore, we will consult experts within the field.

#### 3.2.2 Search strategy

((ABI or ‘acute brain injury’) or (TBI or ‘traumatic brain injury’) or (SAH or aSAH or ‘subarachnoid hemorrhage’ or ‘subarachnoid haemorrhage’) or (ICH or ‘intracerebral haemorrhage’ or ‘intracerebral hemorrhage’)) or (‘stroke’) or (‘ischaemic brain injury’ or ‘ischemic brain injury’) or (‘hypoxic brain injury’ or ‘hypoxic‐ischaemic brain injury’ or ‘hypoxic‐ischemic brain injury’) or (‘anoxic brain injury’ or ‘anoxic‐ischaemic brain injury’ or ‘anoxic‐ischemic brain injury’) and (glucose or glycemia or glycaemia).

### 3.3 Criteria for considering studies for inclusion

#### 3.3.1 Condition or domain being studied

Glycaemic control in patients suffering from acute severe brain injury comprising the following diagnoses: Traumatic brain injury, spontaneous subarachnoid haemorrhage, spontaneous intracerebral haemorrhage, acute ischaemic stroke and anoxic brain injury in need of stay in an intensive care unit.

#### 3.3.2 Participants/population

Patients ≥16 years with acute severe brain injury as defined above, who are treated in an intensive care unit. A study with both adults and children will be included if ≥80% of the study participants are above the age of 15 years or it is possible to retrieve the individual data from the investigators.

#### 3.3.3 Intervention and exposure

Glycaemic control including treatment with glucose or insulin at any dose, formulation and of any duration.

Trials investigating glucose or insulin administration for procedural purposes will be excluded.

#### 3.3.4 Comparator(s)/control

Placebo, ‘active placebo’, ‘no intervention’ or conventional treatment used as standard of care in the ICU, including another level of plasma glucose. Co‐interventions will be allowed if they are distributed equally in both groups.

#### 3.3.5 Types of studies to be included

For the primary and secondary outcomes: We will search for and include randomised clinical trials for the assessments of benefits and harms, irrespective of reported outcomes, publication date, publication language, publication type and publication status.

We will also include quasi‐randomised studies and observational studies that are identified during our searches, for separate analysis of harms. Such studies will be reported narratively and separately. We are aware that primarily including randomised clinical trials will reduce our chance of discovering harms, especially long term and very rare harms resulting from the intervention.

We will include articles reported in the English language. A list of possibly relevant titles in other languages will be provided as an appendix.

#### 3.3.6 Context

Studies in intensive care units (ICUs) of any type, including neurological/neurosurgical, cardiac/coronary care, surgical, medical, mixed and multidisciplinary ICUs.

#### 3.3.7 Types of outcomes

The following outcomes will be analysed:

#### 3.3.8 Primary outcomes

- All‐cause mortality.
- Proportion of participants with unfavourable functional clinical outcome at maximal follow up.
  We will evaluate functional outcome by any outcome scale possible for assessment of favourable/unfavourable outcome including dichotomised Glasgow Outcome Scale (GOS), Glasgow Outcome Scale—extended (GOSE), a modified Rankin Scale (mRS) or Cerebral Performance Category (CPC). We will define an unfavourable outcome as a GOS score of 1–3, GOSE score of 1–5, an mRS score of 3–6 or a CPC score of 3–5. If one of these scales is used with another distinction between unfavourable and favourable outcomes, we will contact the authors and ask for more granular data that will allow us to re‐categorise the patients according to our definitions. If authors cannot be contacted, or the data are unavailable, we will maintain the definitions that were used by the authors. If other scales than those specifically mentioned above are used, we will define unfavourable vs. favourable outcomes as given by the authors.
- Health‐related quality of life at maximal follow up as measured using any scale that has previously been validated for this outcome.

#### 3.3.9 Secondary outcomes

- Proportion of participants with one or more serious adverse events.
  We will consider an event as a serious adverse event if it fulfils the definition of serious adverse events of the International Conference on Harmonization (ICH) Guidelines (ICH‐GCP 1997),^26^ that is, any event that leads to death; is life‐threatening; requires in‐patient hospitalisation or prolongation of existing hospitalisation; or results in persistent or significant disability, congenital birth or anomaly; and any important medical event that may have jeopardised the patient or required intervention to prevent it. We will consider all other adverse events as non‐serious.
- The proportion of participants with hypoglycaemia as defined by the author (dichotomous).
- ICU length of stay (in days).
- Duration of mechanical ventilation (in days).

#### 3.3.10 Explorative outcomes

- ICP.
- Infections.
- Any neurological relevant outcome defined by the authors and not included in our primary or secondary outcomes including data regarding multimodal neuromonitoring.

#### 3.3.11 Timing of outcome measurement

We will apply no restrictions regarding duration of the intervention or length of follow‐up. We will assess outcome data at the end of the trial follow‐up period (maximal follow‐up).

### 3.4 Data collection and analysis

#### 3.4.1 Selection of studies

All abstracts will be screened by two independent reviewers. Any disagreement between authors will be discussed and if uncertainties remain, the trial will be included for full‐text screening. Full‐text papers will be screened by the two reviewers, independently, and will be selected based on the above‐mentioned criteria. Trials will be classified as included, excluded or uncertain. Trials classified as uncertain will be discussed by the two reviewers and a third reviewer will be consulted if needed. If necessary, the corresponding authors will be contacted for clarification. If individual patient data is not already available in the article, the corresponding authors will be asked to supply data for individual patients suffering from the before mentioned brain injuries. Multiple publications on the same trial will be handled as one trial.

#### 3.4.2 Data extraction

Two independent authors will extract predefined data from the included reports. Extracted data items will include trial characteristics (country and year of publication), trial setting, population (inclusion‐ and exclusion criteria), potential funding bias, intervention, data on the predefined outcome and risk of bias. If any discrepancies in the extraction occur that cannot be resolved, a third author will resolve the issue. Corresponding authors will be contacted for further information if there are issues related to data reporting or we are missing further details. Covidence© (Covidence, Melbourne, Australia) will be used for selection of studies and data extraction.^27^

### 3.5 Assessment of risk of bias in included studies

#### 3.5.1 Risk of bias (quality) assessment

Two authors will assess the included studies for risk of bias using the revised Cochrane risk of bias tool for randomised clinical trials (RoB 2.0) as recommend in The Cochrane Handbook of Systematic Reviews of Interventions.^28^ The tool uses five domains by which bias can be introduced into the trial: (1) Bias arising from the randomisation process; (2) bias due to deviations from intended interventions; (3) bias due to missing outcome data; (4) bias in measurement of the outcome; and (5) bias in selection of the reported result.^29^

For details, see Supporting Information S1 ‘Risk of bias assessment’.

The subgroup analysis (see below) will compare intervention effects of trials at low risk of bias with trials of high risk of bias. Observational studies are included solely to assess potential harms and will not undergo the same formal examination of bias though we are aware of the potential higher risk of selection bias. We will separately report these harms, as information on rare or late occurring adverse events are more likely to be reported.^30^, ^31^

#### 3.5.2 Missing data

In case of important missing data such as number of screened or randomised patients or data regarding the intention‐to‐treat analysis, we will contact the authors of the relevant trials to obtain missing data if individual patient data are not available. We will report if data are missing.

#### 3.5.3 Measures of treatment effect

For the primary outcomes, we will use an intention‐to‐treat analysis to make comparisons where possible. We will prefer intention‐to‐treat data, but if we cannot obtain such data, per protocol data will be used in a sensitivity analysis (see Section 3.5.8). For all dichotomous outcomes we will calculate the relative risk (RR) with 95% confidence interval (CI). In case of zero event trial or group, we will employ continuity correction with the value of 0.01. For continuous outcomes, presumably related to measure health‐related quality of life, duration of ICU‐stay and mechanical ventilation, we will calculate the mean difference (MD) with 95% CI. If multiple different scales have been used, we will consider calculating the standardised mean difference (SMD) with 95% CI. We will report results from both fixed‐effect and random‐effects meta‐analysis. If they show differing results, we will choose the one with the most conservative result, which is the estimate closest to zero effect. If the estimates are similar, we will use the one with the highest *p*‐value as the main result.

For these analyses, we will make use of R (R Core Team, Vienna, Austria), a statistical software program. We will adjust for multiplicity as suggested by Jakobsen et al by adjusting the conventional .05 (alpha‐level) with the value halfway between no correction and the Bonferroni correction.^32^ This would correspond to a *p* value of .033 for the primary outcomes. The *p* values of the secondary and exploratory outcomes are not considered regardless of the results of the analysis.

#### 3.5.4 Unit of analysis issues

We will include and search for randomised clinical trials. Trials using a cross‐over design will not be included.

Trials with multiple intervention groups may be pooled into target threshold groups.

Binary data will be added and combined within a two‐by‐two table. If data are continuous, we will combine data following the formula of the Cochrane Handbook for Systematic Reviews of Interventions.^28^ Where the additional treatment arms are not relevant, these data will not be reproduced. ‘Cluster randomisation’ (such as randomisation by clinician or practice) can be employed in trials. When analysing and pooling clustered data this poses problems. First, authors often fail to account for intraclass correlation (ICC) in clustered studies, leading to a ‘unit of analysis' error, whereby *p* values may become spuriously low, CIs unduly narrow and statistical significance overestimated.^33^

For cluster‐randomised trials, we will calculate the ‘design effect’. This is calculated using the mean number of participants per cluster (*M*) and the ICC [design effect = 1 + (*M* − 1) × ICC]. Where clustering is not accounted for in primary studies, we will seek to contact the first authors of studies to obtain ICCs for their clustered data and to adjust for this by using accepted methods. If the ICC is not reported or obtainable it will be assumed to be 0.1.^33^

#### 3.5.5 Strategy for data synthesis

We will conduct meta‐analysis based on the recommendations in The Cochrane Handbook for Systematic Reviews of Interventions. In order to assess the intervention effect, we will utilise the eight‐step approach proposed by Jakobsen et al.^34^ Meta‐analysis will be performed only when effect measures from two or more trials are comparable. We will address heterogeneity across trials by visual inspection of forest plots and by performing *χ* ^2^ test with a *p* < .10 as statistical level of significance and measuring the quantities of heterogeneity by *I* ^2^ statistics. Furthermore, we will perform subgroup analyses for investigating possible reasons for heterogeneity (see the ‘Subgroup analyses’ section below). According to the Cochrane Handbook for Systematic Reviews of Interventions, heterogeneity is defined as considerable if *I* ^2^ is between 75% and 100%, may represent substantial heterogeneity between 50% and 90%, may represent moderate heterogeneity between 30% and 60%, and might not be important between 0% and 40%.^28^ We may ultimately decide that a meta‐analysis should be avoided if heterogeneity is considerable and the results will be reported narratively. We will report our results according to the PRISMA statement (Supporting Information S2).^35^

#### 3.5.6 Trial Sequential Analysis

Trial Sequential Analysis (TSA) is a method developed by Copenhagen Trial Unit (CTU), which combines information size calculation for a meta‐analysis—or the meta‐analytic sample size—with the threshold for statistical significance and the level of power.^36^, ^37^ For meta‐analysis with sparse data or multiple testing of accumulating data the risk of random errors and imprecision is increased.^32^, ^38^ For dichotomised data a relative risk reduction (RRR), defined at the protocol stage, is used alongside the proportion of events in the control group and the heterogeneity or diversity in the analysis. For continuous outcomes the variance and minimally relevant differences of the outcomes are used instead of RRR and proportion in the control group^39^ As data are cumulated the use of Lan‐DeMets' stopping boundaries in the TSA will enable testing for significance every time a new trial is introduced in the meta‐analysis. Based on the required information size, trial sequential monitoring boundaries for benefit, harm and futility can be constructed to determine the statistical inference concerning cumulative meta‐analysis that has not yet reached the required information size.^36^ If the cumulative *Z*‐curve crosses the boundaries of harm or benefit firm evidence can be established without reaching the required information size and further trials may be redundant. If the boundaries are not surpassed it is possible to conclude that more trials are needed before a certain intervention effect can be established. Firm evidence of a lack of intervention effect is possible to conclude if the cumulative *Z*‐curve crosses the area of futility.

We will do a Trial Sequential Analysis using the most updated version of the TSA software developed by CTU to evaluate the risk of random errors due to repetitive testing in a cumulative meta‐analysis. Also, we want to estimate the required information size based on the observed number of patients with an outcome in the control group and the alpha adjusted CI for a better interpretation of imprecision in the analysis and for evaluation of GRADE.

For the dichotomous outcome, all‐cause mortality, we will conduct the Trial Sequential Analysis using a relative risk reduction (RRR) of 15%. 15% RRR has been chosen as a similar effect was shown in the randomised controlled study by the NICE SUGAR investigators, who found an OR at 1.14 (CI 1.02–1.28; *p* = .02) for mortality favouring conventional glucose‐control.^12^ We have chosen a conservative estimate of an RRR of 20% for proportion of patients with unfavourable outcomes. For quality of life, we will use the observed standard deviation (SD) from the included trials to calculate the minimally relevant difference (SD/2) and the variance (SD*SD). We will adjust the alpha‐level (.033) and beta level (10%) for all primary outcomes and use the proportion of events found in the control group.

We will consider secondary outcomes as explorative but we will still adjust the significance level for the Trial Sequential Analysis (*p* value .0025) to avoid an increase in the family‐wise error rate as four outcomes are analysed.^32^

For secondary dichotomous outcomes an RRR of 20% will be used and a beta of 90%. For continuous outcomes we will use the observed SD from the trials to calculate a minimally relevant difference as SD/2 and the variance (SD*SD). All TSA will be adjusted using diversity from the included trials instead of inconsistency.

Trial Sequential Analysis will not be conducted on the exploratory outcomes ICP, infections or any neurological relevant outcome.

#### 3.5.7 Network meta‐analysis

Potentially, we will do an explorative network metanalysis for the primary outcomes if the included material has the necessary amount of interventions to divide groups into three or more different glycaemic target‐groups.^28^

A potential network meta‐analysis will be performed using R statistics with the package ‘netmeta’.

#### 3.5.8 Sensitivity analysis

We will conduct a sensitivity analysis to assess the potential impact of missing outcome data. We will perform sensitivity analyses on our primary and secondary outcomes with ‘best‐to‐worst‐case’ and ‘worst‐to‐best‐case’ scenarios if patients are lost to follow up or if we only have access to per protocol data. We will report the results of these scenarios in our systematic review. If heterogeneity is present, it is possible that other post‐hoc sensitivity analyses can be necessary to elucidate the causes of heterogeneity.

#### 3.5.9 Analysis of subgroups or subsets

In order to assess whether a given effect is present in only a subgroup of trials, patients or dosage regimens, or whether the effect is influenced by the type of comparator or heterogeneity within a group of trials, we will conduct the following subgroup analyses if made possible by the obtained data:

- Comparing estimates of the pooled intervention effect in trials at overall ‘low risk of bias’ to that of trials at overall ‘high risk of bias’.
- Comparing a restrictive glucose control regime to a liberal glucose control where restrictive is defined as a regime targeting ≤8.2 mmol/L (147 mg/dL) and liberal is defined as a regime targeting ≥8.3 mmol/L (150 mg/dL).
- Comparing a restrictive glucose control regime to a liberal glucose regime defined by authors in each included trial.
- Comparing estimates of the pooled intervention effect according to patient diagnosis, that is, comparing aneurysmal subarachnoid haemorrhage, traumatic brain injury, acute ischaemic stroke, intracerebral haemorrhage, hypoxic and anoxic brain injury to each other.

#### 3.5.10 Assessment of the overall quality of evidence

We will assess the quality of evidence across studies according to the Grading of Recommendations, Assessment, Development and Evaluation (GRADE) methodology recommendations for our three primary outcomes (all‐cause mortality, health‐related quality of life and severe adverse events) and present this in a summary of findings table. We will use the five GRADE considerations (risk of bias in the trials, consistency of effect, imprecision, indirectness and publication bias).^32^, ^40^

For imprecision we will use the Trial Sequential Analysis. If the accrued information size is lower than 50% of the required information size, we will downgrade two levels, and one level if it is between 50% and 100%. We will not downgrade imprecision if the cumulative *Z*‐curve breaches the monitoring boundaries of benefit, harm, futility or if the diversity adjusted required information size has been reached. All decisions to downgrade will be justified in the summary of findings table.

Two review authors will independently judge the certainty of the evidence with disagreement resolved by discussion or involving a third review author.

#### 3.5.11 Possible addition to this systematic review protocol

If we receive individual patient data (IPD) from the included trials, we intend to use intention‐to‐treat data. Furthermore, we will consider doing an additionally IPD meta‐analysis of the raw data if an IPD meta‐analysis can increase precision and reliability.

We will report an additional IPD meta‐analysis according to the PRISMA‐IPD.^41^

#### 3.5.12 Differences between the protocol and the review

We will conduct a systematic review according to this published protocol and report any deviations in a section of ‘Differences between protocol and review’ in the systematic review.

If we need to amend this protocol, we will give the date of each amendment, describe the changes and give the rationale in this section. Changes will not be incorporated into the protocol.

## 4 DISCUSSION

This systematic review with meta‐analyses and Trial Sequential Analysis aims at comparing and explore benefits and harms of restricted versus liberal blood glucose control in patients with severe acute brain injury admitted to an intensive care unit.

These patients are at high risk for a poor outcome and it is possible that the outcome for these patients can be improved.

Primary outcome will be all‐cause mortality, functional outcome and health‐related quality of life. Secondary outcomes will be serious adverse events, hypoglycaemia, length of stay in the ICU and duration of mechanical ventilation. Additionally, we will assess exploratory outcomes such as ICP and infections.

This protocol has several strengths. It has a predefined methodology based on the Cochrane Handbook for Systematic Reviews of interventions and the GRADE assessments. We also intend to use the eight‐step assessment by Jacobsen et al., the PRISMA statement and perform a trial sequential analysis. This protocol takes the risks of random errors and systematic errors under consideration and we will contact all trial authors regarding individual patient data and address potential missing data.

The protocol does hold limitations as well. It is possible there is heterogeneity in‐between the glucose‐control regimes of the existing trials' and, therefore, it is not relevant to do a meta‐analysis. Data analysis can be limited if it is impossible to obtain missing data from the authors or individual patient data. Also, focussing primarily on randomised clinical trials increases the risk of finding more benefits than harms. Though, we will include observational studies for observations of harms.

Overall, we intend to do a thorough systematic review and hopefully the achieved results can be useful in planning future randomised trials of good quality and make basis for decisions in clinical practise based on evidence.

## AUTHOR CONTRIBUTIONS

Anne‐Sophie Worm Fenger, Markus Harboe Olsen, Maria Louise Fabritius, Christian Gunge Riberholt and Kirsten Møller designed the study; all authors critically revised the protocol and approved the final version. Anne‐Sophie Worm Fenger is the guarantor of the systematic review.

## FUNDING INFORMATION

No specific funding was received for this project. The project has been initiated by AF and Professor Kirsten Møller (sponsor), both from Department of Neuroanaesthesiology, Neuroscience Centre, Rigshospitalet, University of Copenhagen.

## CONFLICT OF INTEREST

The authors declare no conflicts of interest.

## Supporting information


**Data S1** Risk of bias assessment

**Data S2** PRISMA‐P checklist
