# Supplementary material for: Glycaemic control for patients with severe acute brain injury: Protocol for a systematic review
Source: Acta Anaesthesiol Scand. 2022 Nov 12;67(2):240–7. doi: 10.1111/aas.14166 (PMC10099998; doi:10.1111/aas.14166)
Supplement: Supplementary file 1 — Data S1 Risk of bias assessment [file AAS-67-240-s002.docx]

**Supplemental material**

**Risk of bias assessment**

**Randomisation process**

Low risk of bias: If the randomisation process is well described, random, and the allocation sequence was concealed.

High risk: Missing randomisation, if the randomisation process is undescribed or unconcealed, or baseline characteristics between intervention groups suggest a problem with the randomisation process.

**Deviations from intended interventions**

Low risk: Participants, carers, and personnel are unaware of intervention groups during the trial or participants, carers, and personnel were aware of intervention groups during the trial, but any deviations from intented intervention were unlikely to impact on the outcome and no participants were analysed in the wrong intervention groups.

High risk: No description of deviations, how deviations are handled in the trial analysis, or the deviations are unbalanced between intervention groups and likely to have affected the outcome, or some participants were analysed in another group than their received intervention.

**Missing outcome data**

Low risk of bias: If a trial has a published protocol with defined outcomes before the trial was initiated and the published study have published the predefined outcomes. In case of no protocol published, the reporting of all-cause mortality and serious adverse events.

High risk of bias: If a study has a published protocol and the reported outcomes pre-specified are not reported, or an unclear or high degree of missing data if there are a different proportion or different reasons for missing data in the two groups and there is no evidence that the effect estimate is robust to missing data.

**Measurements of the outcome**

Low risk: If the measurement of the outcome is reported and known as a valid marker of the given outcome.

High risk: If the method of measurement is unvalidated for the given outcome, or the assessors of the effect were aware of the intervention.

**Selection of the reported result**

Low risk: The results from a trial was analysed according to a pre-specified plan and the results are reasonable on basis of the data analyses.

High risk: If reported outcome data are likely to have been selected, or the pre-specified plan has changed.

**Overall risk of bias**

A trial will be classified as overall “low risk of bias” if all five bias domains are classified as “low risk of bias”.

A trial with any domain classified as “high risk of bias” will be classified as “high risk of bias”.
