# Supplementary material for: Glycaemic control for patients with severe acute brain injury: Protocol for a systematic review
Source: Acta Anaesthesiol Scand. 2022 Nov 12;67(2):240–7. doi: 10.1111/aas.14166 (PMC10099998; doi:10.1111/aas.14166)
Supplement: Supplementary file 2 — Data S2 PRISMA‐P checklist [file AAS-67-240-s001.docx]

**PRISMA-P (Preferred Reporting Items for Systematic review and Meta-Analysis Protocols) 2015 checklist: recommended items to address in a systematic review protocol***

| Section and topic | Item No | Checklist item |
| --- | --- | --- |
| ADMINISTRATIVE INFORMATION | | |
| Title: |  |  |
| Identification | 1a | Page 1 |
| Update | 1b | - |
| Registration | 2 | The protocol is not registered yet. The plan is to register at PROSPERO. |
| Authors: |  |  |
| Contact | 3a | Page 1 |
| Contributions | 3b | Page 18 |
| Amendments | 4 | Page 17 |
| Support: |  |  |
| Sources | 5a | Page 18 |
| Sponsor | 5b | Page 18 |
| Role of sponsor or funder | 5c | Page 18 |
| INTRODUCTION | | |
| Rationale | 6 | Page 4-5 |
| Objectives | 7 | Page 5 |
| METHODS | | |
| Eligibility criteria | 8 | Page 6-7 |
| Information sources | 9 | Page 5-6 |
| Search strategy | 10 | Page 6 |
| Study records: |  |  |
| Data management | 11a | Page 9-11 |
| Selection process | 11b | Page 9-11 |
| Data collection process | 11c | Page 9-11 |
| Data items | 12 | Page 11 |
| Outcomes and prioritization | 13 | Page 8-9 |
| Risk of bias in individual studies | 14 | Page 10-11 + S1 File. Risk of bias assessment |
| Data synthesis | 15a | Page 12-13 |
|  | 15b | Page 13 |
|  | 15c | Page 13 |
|  | 15d | Pag 12-15 |
| Meta-bias(es) | 16 | Page 16-17 |
| Confidence in cumulative evidence | 17 | Page 17 |

*** It is strongly recommended that this checklist be read in conjunction with the PRISMA-P Explanation and Elaboration (cite when available) for important clarification on the items. Amendments to a review protocol should be tracked and dated. The copyright for PRISMA-P (including checklist) is held by the PRISMA-P Group and is distributed under a Creative Commons Attribution Licence 4.0.**

*From: Shamseer L, Moher D, Clarke M, Ghersi D, Liberati A, Petticrew M, Shekelle P, Stewart L, PRISMA-P Group. Preferred reporting items for systematic review and meta-analysis protocols (PRISMA-P) 2015: elaboration and explanation. BMJ. 2015 Jan 2;349(jan02 1):g7647.*
